# Southern Ocean contribution to both steps in deglacial atmospheric CO_2_ rise

**DOI:** 10.1038/s41598-021-01657-w

**Published:** 2021-11-11

**Authors:** Thomas A. Ronge, Matthias Frische, Jan Fietzke, Alyssa L. Stephens, Helen Bostock, Ralf Tiedemann

**Affiliations:** 1 Alfred-Wegener-Institut Helmholtz-Zentrum für Polar- und Meeresforschung, Am Alten Hafen 26, 27568 Bremerhaven, Germany; 2 GEOMAR Helmholtz-Zentrum für Ozeanforschung, Kiel, Germany; 3 IODP International Ocean Discovery Program, College Station, USA; 4 The University of Queensland, Brisbane, Australia

**Keywords:** Biogeochemistry, Climate change, Ocean sciences, Palaeoceanography, Palaeoclimate

## Abstract

The transfer of vast amounts of carbon from a deep oceanic reservoir to the atmosphere is considered to be a dominant driver of the deglacial rise in atmospheric CO_2_. Paleoceanographic reconstructions reveal evidence for the existence of CO_2_-rich waters in the mid to deep Southern Ocean. These water masses ventilate to the atmosphere south of the Polar Front, releasing CO_2_ prior to the formation and subduction of intermediate-waters. Changes in the amount of CO_2_ in the sea water directly affect the oceanic carbon chemistry system. Here we present B/Ca ratios, a proxy for delta carbonate ion concentrations Δ[CO_3_^2−^], and stable isotopes (δ^13^C) from benthic foraminifera from a sediment core bathed in Antarctic Intermediate Water (AAIW), offshore New Zealand in the Southwest Pacific. We find two transient intervals of rising [CO_3_^2−^] and δ^13^C that that are consistent with the release of CO_2_ via the Southern Ocean. These intervals coincide with the two pulses in rising atmospheric CO_2_ at ~ 17.5–14.3 ka and 12.9–11.1 ka. Our results lend support for the release of sequestered CO_2_ from the deep ocean to surface and atmospheric reservoirs during the last deglaciation, although further work is required to pin down the detailed carbon transfer pathways.

## Introduction

**Figure 1:**
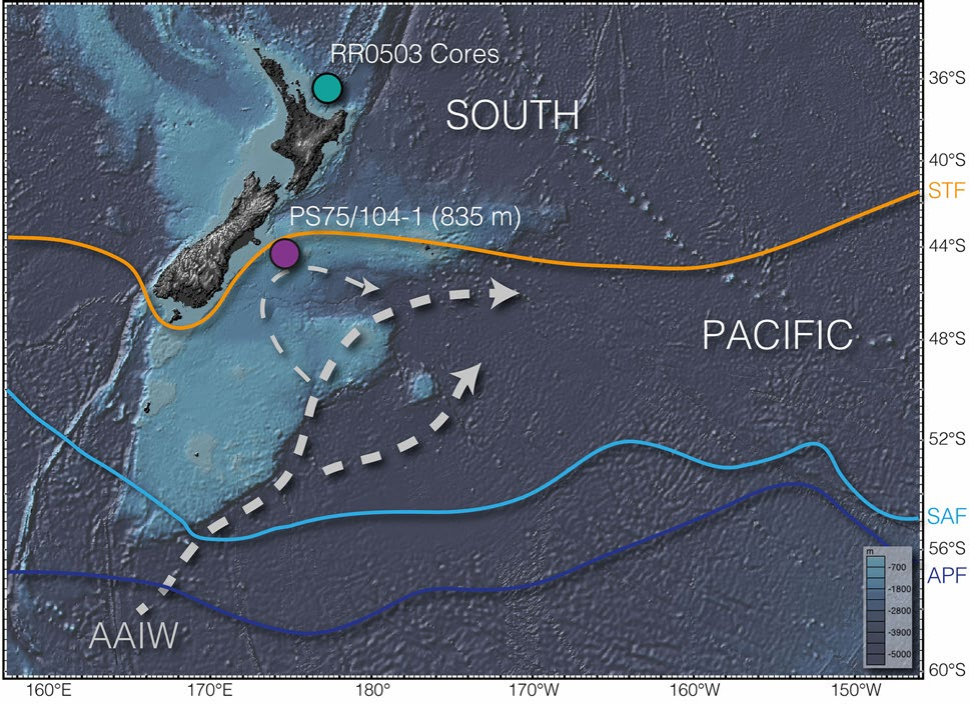
Schematic view of Southwest Pacific research area and oceanographic features important to this study. Purple dot—location of PS75/104-1; teal dot Bay of Plenty core locations used by Allen et al.^39,52^; *AAIW* Antarctic intermediate water; orange line—STF/subtropical front; light blue line—SAF/subantarctic front; dark blue line—APF/Antarctic polar front. Fronts according to Orsi et al.^71^. AAIW path (grey arrows) according to Bostock et al.^12^. Map created with GeoMapApp version 3.6.12 (https://www.geomapapp.org).

On glacial-interglacial timescales, the carbon cycle is profoundly linked to the global Thermohaline Circulation or Atlantic Meridional Overturning Circulation (AMOC), with small changes in the capacity of oceanic carbon sequestration resulting in major changes of atmospheric CO_2_-levels. Strong support for this process comes from the co-evolution of deglacial changes in ocean carbon with atmospheric CO_2_^1^, Δ^14^C (corrected ^14^C activity)^2^, and atmospheric δ^13^C-values^3^. These patterns indicate that vast amounts of CO_2_ were released from a reservoir (or reservoirs) with low ^14^C and δ^13^C values during the last deglaciation. Evidence for the existence of such reservoirs has been found in all sectors of the Southern Ocean^4–8^, the North Pacific^9^, as well as northern permafrost soils^10,11^. However, evidence for the pathway of sequestered carbon from the deep-waters to the surface and ultimately the atmosphere is limited. Upwelling of Circumpolar Deep Waters (CDW) in the Antarctic and Subantarctic Zones of the Southern Ocean is the most likely pathway for the carbon-rich waters to debouch carbon to the atmosphere. These upwelled waters are subsequently subducted and exported northward as intermediate- and mode-waters (Fig. 1).

Any change in the sequestration and cycling of CO_2_ in the ocean will affect the marine inorganic carbon system. In this respect, reconstructions of water mass carbon chemistry (carbonate ion concentrations; [CO_3_^2−^]) can yield important insights into the effect of past changes in dissolved inorganic carbon (DIC), and the oceanic carbon reservoir.

Here we present reconstructions of [CO_3_^2−^] and δ^13^C on benthic foraminifers (*Cibicidoides wuellerstorfi* and C. *dispars*) from an intermediate-water record recovered at 44.4°S offshore New Zealand (PS75/104–1; 835 m; Fig. 1). PS75/104-1 is bathed by Antarctic Intermediate Water (AAIW)^12^, and thus changes in the [CO_3_^2−^] will reflect changes in DIC that could provide clues to air-sea gas exchange occurring upstream in the Southern Ocean prior to the subduction of these waters to intermediate depths. Our study presents evidence for deglacial changes in the Pacific DIC pool, highlighting a pathway of sequestered CO_2_ from the ocean to the atmosphere during Heinrich Stadial 1 (HS1; ~ 18–14.5 thousand years before present (ka)) and the Younger Dryas (YD; ~ 12.9–11.6 ka).

## Results

**Figure 2:**
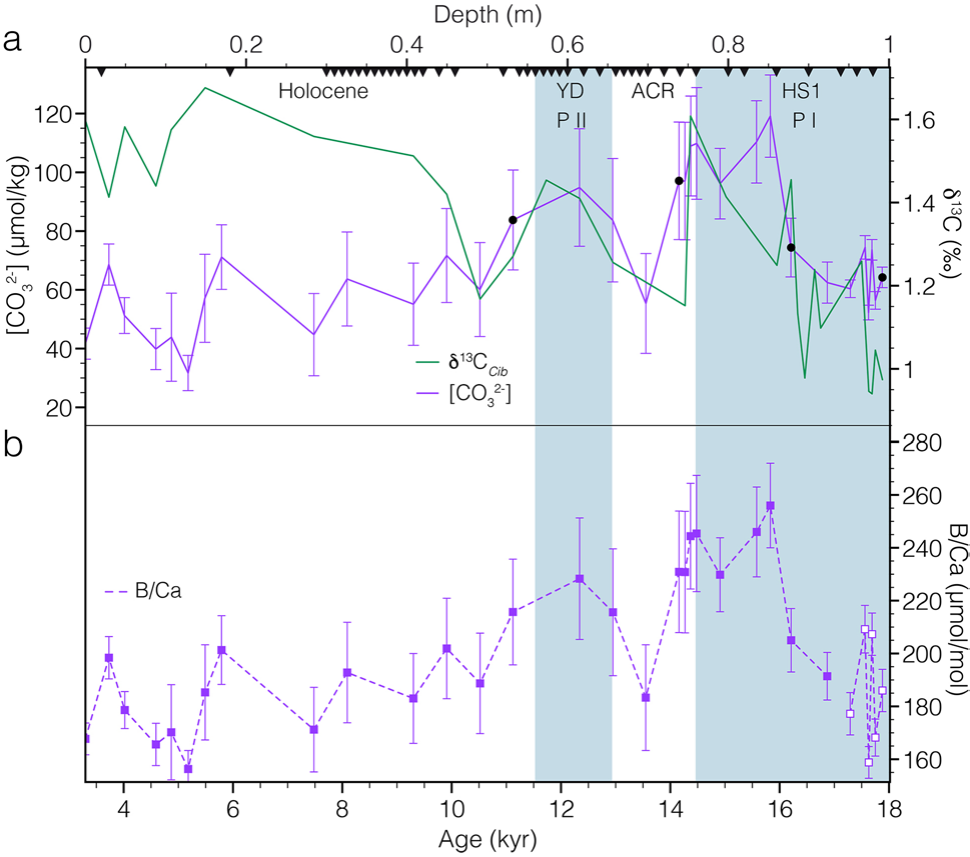
**(a)** δ^13^C (green) and carbonate ion data [CO_3_^2−^] (purple) of core PS75/104-1. Black dots indicate samples that might be affected by bioturbation. Black triangles—radiocarbon dates^8,13^. Error bars—1σ. **(b)** Uncalibrated B/Ca data of PS75/104-1. Filled symbols—*C. wuellerstorfi*; empty symbols—*C. dispars*. Blue shading—*HS1* Heinrich Stadial 1, *YD* Younger Dryas, *ACR* Antarctic Cold Reversal. P I/II—CO_2_ pulses as shown in Fig. 3.

Site PS75/104 is located about 150 km to the east of the South Island of New Zealand, south of the Subtropical Front (Fig. 1). We conducted B/Ca and stable carbon isotope measurements on benthic foraminifers to infer past AAIW changes in carbon chemistry of the past ~ 22 thousand years (kyr). The age model was developed, using a high resolution ^14^C-based record of 56 individual dates^13^. Age uncertainties reported by Küssner et al.^13^ range between 15 and 190 years and are too small to show in Fig. 2. Thus, we have high confidence in the timing of the records reported here. While four samples might be affected by *Zoophycos* bioturbation^13^ (Fig. 2), excluding these samples does not impact our age model, the general trends seen in our proxy records, or our interpretations.

During the late glacial and early deglaciation (18–17 ka) B/Ca values measured on *C. dispars* show a high variability, averaging at ~ 184 µmol/mol, similar to the nearest measurement on *C. wuellerstorfi* of 191.4 µmol/mol (Fig. 2), confirming that *C. dispars* can be used in the analysis of B/Ca. During the early deglacial (HS1), B/Ca values increase until ~ 14.3 ka, reaching a maximum of 256 µmol/mol at 15.8 ka. During the Antarctic Cold Reversal (ACR) values decrease to 183.3 µmol/mol (similar to the glacial values). A second significant increase to about 228.3 µmol/mol occurs between ~ 12.9 and 11.1 ka, followed by a progressive, yet variable decrease within the Holocene (Fig. 2).

Carbonate ion concentrations of core PS75/104-1 range from 31.6 µmol/kg up to 119.1 µmol/kg (Fig. 2). The most pronounced increase in [CO_3_^2−^] (60.3–109.8 µmol/kg) occurred between 17.2 and 14.5 ka and is paralleled by an equally pronounced rise in δ^13^C benthic from ~ 1.25 to 1.6‰. Both records display a second, yet less pronounced, increase between 13.55 and 10.52 ka (Fig. 2). During the Holocene, both records diverge, with increasing δ^13^C and decreasing [CO_3_^2−^]. Following the YD disturbance to the inorganic carbon system, carbonate compensation drives back the system back to its original state^14^, while δ^13^C continues to increase^15^.

## Discussion

**Figure 3:**
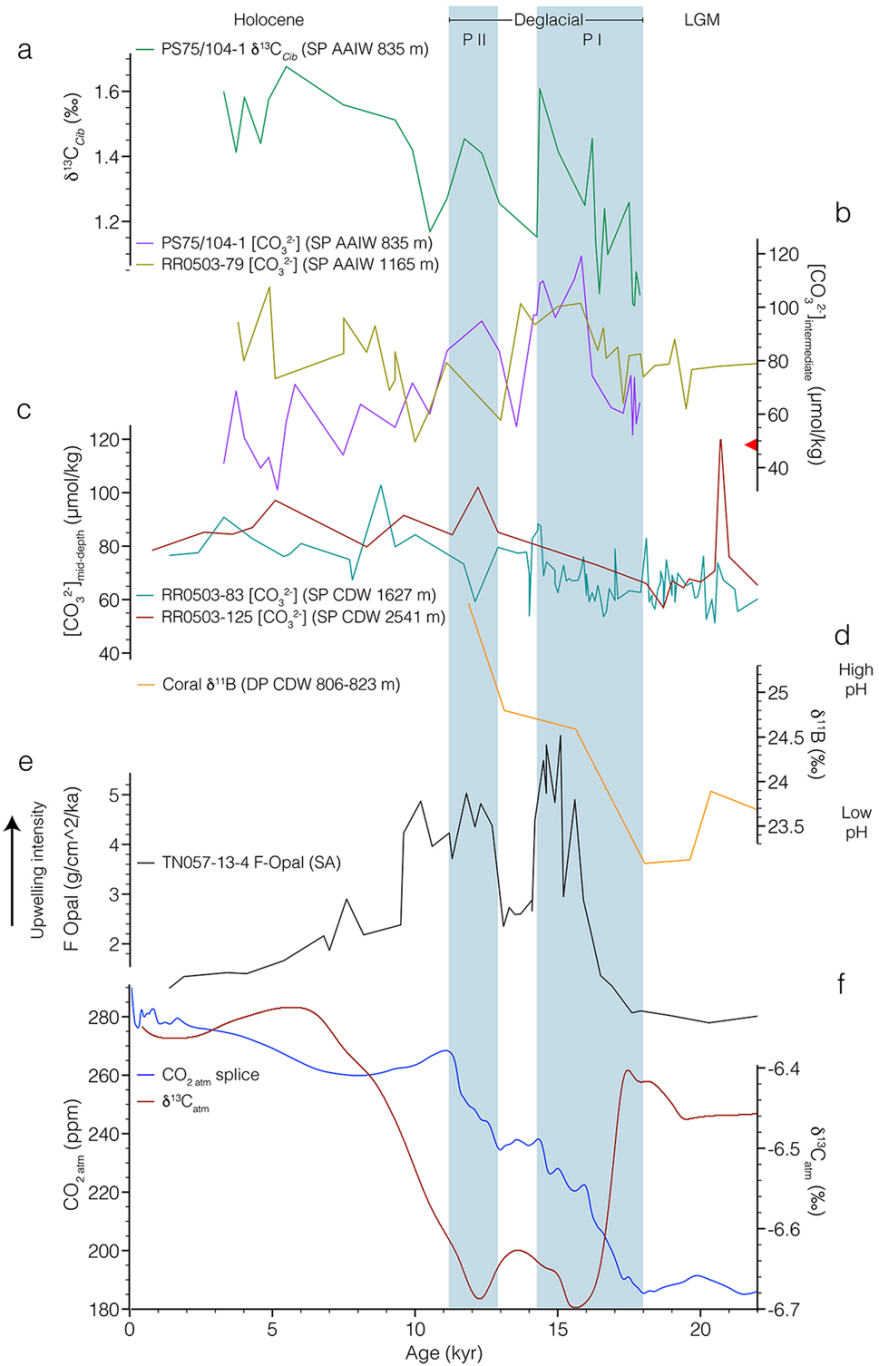
Southern Ocean proxy records in relation to atmospheric records. **(a)** PS75/104-1 δ^13^C (green). **(b)** Southwest Pacific intermediate-water [CO_3_^2−^] records. PS75/104-1 (purple; this study), RR0503-79^39^ (yellow). Red triangle—modern [CO_3_^2−^]_sat_ 49.48 µmol/kg at depth of PS75/104-1^58^. **(c)** Southwest Pacific mid depth [CO_3_^2–^] records. RR0503-83 (teal), RR0503-125^39^ (dark-red). **(d)** Drake Passage coral δ^11^B data^34^ (orange). **(e)** South Atlantic upwelling and opal flux^50^ (black). **(f)** Atmospheric δ^13^C^3^ (maroon) and CO_2_ splice^1^ (blue). Blue shading—deglacial pulses in atmospheric CO_2_. *P I* first pulse—Heinrich Stadial 1, *P II* second pulse—Younger Dryas, *SP* South Pacific, *SA* South Atlantic, *DP* drake passage.

Changes in Southern Ocean deep-water ventilation are often used to explain the two deglacial pulses of rising atmospheric CO_2_^5,7,8,16,17^ (Fig. 3). However, while being an indicator for deep-water residence time, radiocarbon alone does not allow for the direct analysis of the past oceanic carbon pool and changes in DIC. Barring lateral water mass transport, the primary driver for changes in δ^13^C_DIC_ and [CO_3_^2−^] is the biological pump, the export of organic matter from the surface into deeper water masses, and its subsequent degradation is a primary driver for changes in δ^13^C_DIC_ and [CO_3_^2−^]^14,18^. With progressive export of carbon (CO_2_), the biological carbon pump increases the DIC content of a given water mass, while decreasing its δ^13^C and [CO_3_^2−^] values. Depending on the balance between CO_2_ sequestration via the biological carbon pump, circulation and ventilation via the upwelling of deep water masses, the circumpolar Southern Ocean can alter between a carbon sink or source.

**Figure 4:**
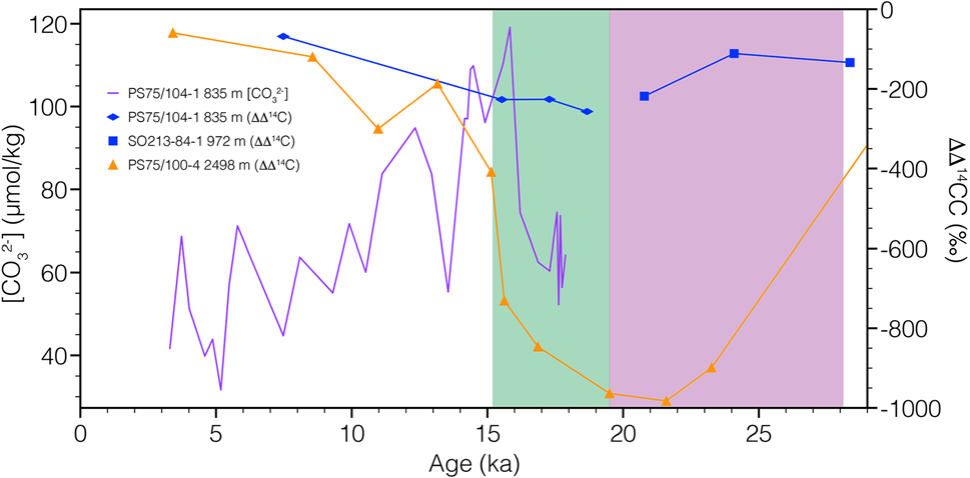
PS75/104-1 [CO3^2−^] (purple line) vs. intermediate and deep-water ΔΔ^14^C^15^. Blue diamonds—PS75/104-1; blue squares—SO213-84-1; orange triangles—PS75/104-1. Purple and green shading—glacial and early deglacial interval of potential CO_2_-release from pockmarks^26^.

Several processes might have resulted in the pronounced transient increase in PS75/104-1 [CO_3_^2−^] and δ^13^C during HS1 (Fig. 3), such as increased surface export, release of carbon-rich fluids from pockmarks, or the discharge of CO_2_ from an oceanic carbon reservoir that built-up during the preceding glacial and fed into the formation area of SW Pacific AAIW. In this case the area, where surface waters are influenced by both the upwelling of deep waters south of the APF, and then air-sea exchange processes as it flows north due to Ekman transport and subducted as intermediate waters. During glacial times, a combination of multiple climatic factors enhanced the ability of the Southern Ocean to sequester CO_2_. Lower surface temperatures allowed for increased uptake of CO_2_^19^, while higher fluxes of iron-rich dust^20^ resulted in increased primary productivity as a result of iron fertilization of the nutrient-rich Subantarctic Sector of the Southern Ocean^21^. This led to enhanced export of carbon to the deep ocean via the biological pump in the Atlantic sector. However, there is no clear evidence of increased productivity in the Subantarctic Sector of the SW Pacific^22–25^, while it was decreased in the Antarctic Sector^23,24^. ^230^Th fluxes of biogenic matter in our research area have shown no significant local change in export production off New Zealand since the LGM^25^. Thus, we assume that changes in productivity and surface export did not play a dominant role in driving SW-Pacific AAIW [CO_3_^2−^] at our core site. Yet, as AAIW chemistry is also influenced by regional, zonal, and meridional processes, we acknowledge the further need for additional investigations to test the feasibility of our interpretation. The distinct anti-phased pattern of atmospheric δ^13^C (Fig. 3f)^3^ and PS75/104-1 suggests that AAIW δ^13^C is not driven by the atmosphere via air-sea gas exchange. An additional factor that potentially influenced water mass carbon chemistry in the SW Pacific might have been the release of CO_2_ from pockmarks that are documented in the research area^26^. During the late glacial and early deglacial, carbon-rich fluids were probably released from the seafloor off New Zealand and might have contributed to extremely low CDW and AAIW ^14^C values^8,26^. The injection of CO_2_ would also affect [CO_3_^2−^] of their respective water masses (Fig. S1). However, as the records of PS75/104-1 ^14^C^8^ does not point toward a deglacial influence of ^14^C-dead CO_2_ from pockmarks^26^ (Fig. 4) and as the transient increase in [CO_3_^2−^] is interpreted as a loss of CO_2_ from a water mass^14,27^, we expect that the release of carbon-rich fluids did not play a role in the evolution of AAIW [CO_3_^2−^] during the time interval covered by our study.

The expansion of Antarctic sea ice toward the north^28–30^, a displacement of Southern Westerly Winds^24,31^, and increased stratification^15,32–34^, reduced deep-water ventilation and circulation^5,7,8^, reduced upwelling and release of CO_2_ in the Polar Frontal Zone, all allowed for the accumulation of carbon in the deep glacial Southern Ocean. Reconstructions of deep-water [CO_3_^2−^] show that the deep South Atlantic was ~ 15 µmol/kg lower and stored ~ 30 gigatons of additional carbon during the Last Glacial Maximum (LGM) than during the Holocene^35–37^.

**Figure 5:**
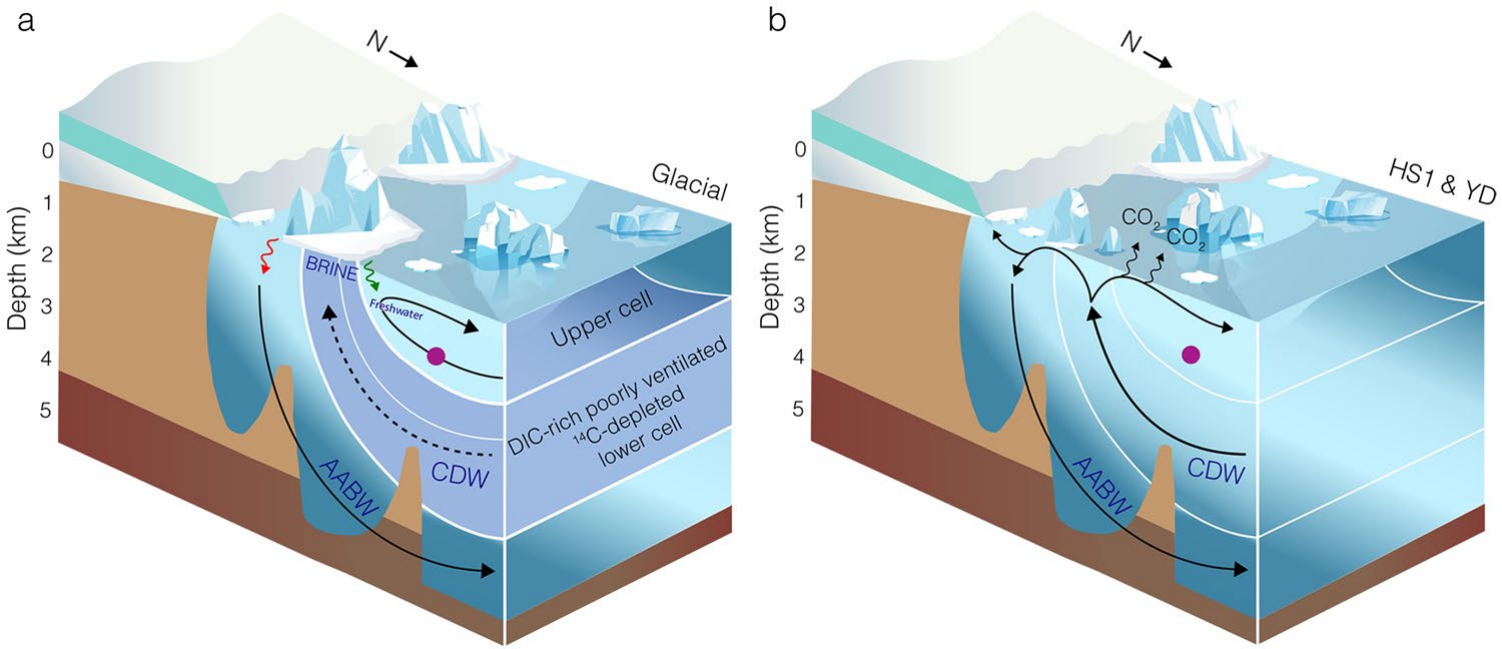
Schematic South Pacific overturning and carbon cycling. **(a)** Last Glacial Maximum. Sluggish CDW circulation with separated lower and upper cells. Lower DIC-rich cell with very depleted ΔΔ^14^C values^8^. **(b)** Heinrich Stadial 1 (HS1) and Younger Dryas (YD) scenario. Progressive increase in deep-water overturning results in the release of CO_2_ from DIC-rich and high pCO_2_ CDW. This release causes the transient rises in AAIW [CO_3_^2−^] observed in downstream PS75/104-1 indicated by purple dot. Green arrow—input of freshwater into the upper cell^15^, red arrow—input of highly saline brine into the lower cell^33^.

**Figure 6:**
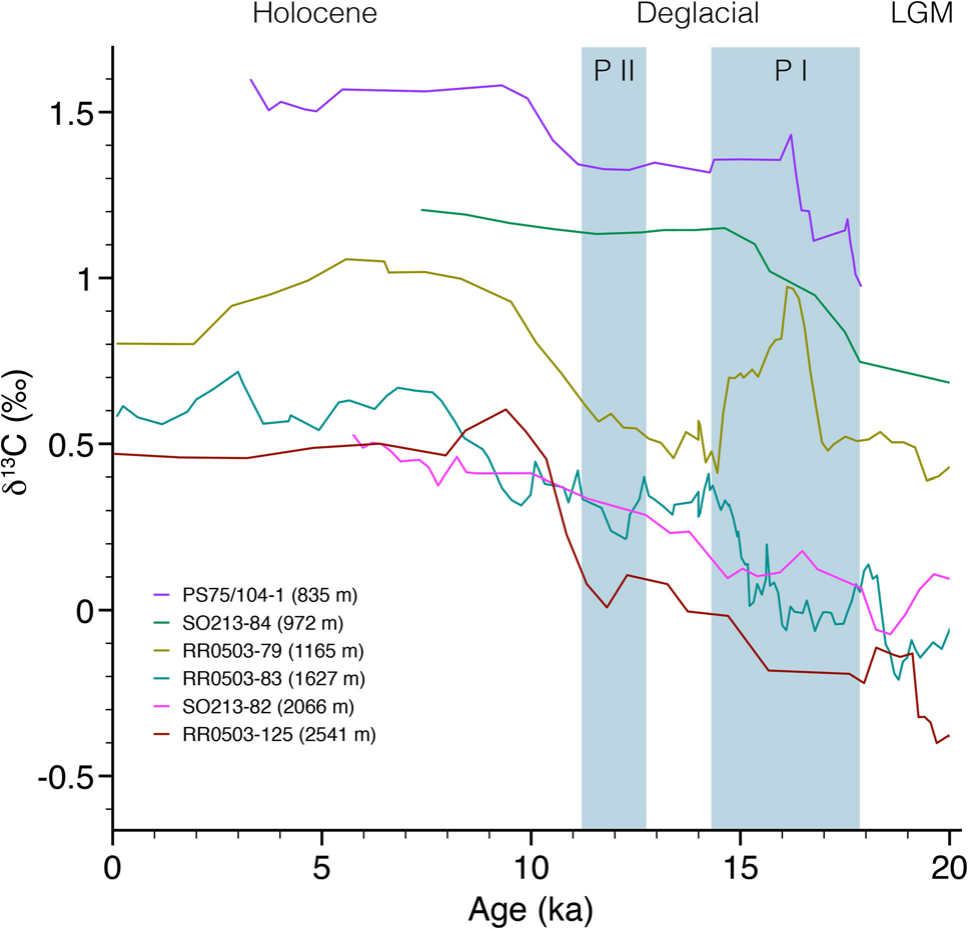
Intermediate- to deep-water δ^13^C records (four-point running average) from the Bay of Plenty (RR0503 cores)^17^ and Chatham Rise^8^. The pattern and general trends of the shallowest core PS75/104-1 (this study) are very similar to Chatham Rise core SO213-84-1^15^ and Bay of Plenty record RR0503-79^17^. Blue shading—deglacial pulses in atmospheric CO_2_. *P I* first pulse—Heinrich Stadial 1, *P II* second pulse—Younger Dryas.

HS1 was marked by the most pronounced increase in atmospheric CO_2_^1^ and was paralleled by a similar increase in PS75/104-1 [CO_3_^2−^] of ~ 58 µmmol/kg (Figs. 2 and 3). Transient increases in [CO_3_^2−^], as recorded by PS75/104-1, reflect a loss of CO_2_ and the subsequent return to the previous state of the marine carbon system via carbonate compensation^14,27^. The inverse relation of [CO_3_^2−^] and water mass pCO_2_^38^, imply that the observed [CO_3_^2−^] rise is consistent with the HS1 release of CO_2_ via the Southern Ocean (Fig. 5b). The release of sequestered CO_2_ is furthermore supported by our record of benthic δ^13^C that closely parallels the increase in observed [CO_3_^2−^] as well as the patterns observed in AAIW RR0503-79^17,39^ (Figs. 3 and 6) and Bay of Plenty δ^18^O and δ^13^C gradients^40^. This trend is in good agreement with the loss of metabolic (high ^12^C) CO_2_ via air-sea gas exchange in the formation area of AAIW^40^ (Fig. S1). However, other factors such as the thermodynamic effect, an overprint of the atmospheric signal^41^, changes in export production (EP), and changes in AAIW formation should be considered when interpreting our records. Air sea exchange under colder temperatures shifts the δ^13^C values toward higher values^42–44^. Thus, warming temperatures tend to shift the system in the direction of higher δ^13^C values^39^. Our HS1 and YD trends in δ^13^C and [CO_3_^2−^] (Fig. S1) are more in line with the slope predicted for regenerated organic carbon^39^, and thus imply that carbon sequestration via the biological pump, and release via ventilation was the more dominant driver. Another process that was observed to have a pronounced influence, is the overprint of atmospheric δ^13^C values on surface and recently ventilated waters^41^. Several δ^13^C-records follow the atmospheric pattern, while minor differences can be attributed to different temperatures during air-sea exchange^41^. In contrast to these records, PS75/104-1 δ^13^C is antiphased to the atmospheric record^3^ during HS1 and the YD (Fig. 3). During the Holocene, however, we observe a strong correlation between atmospheric and AAIW δ^13^C values that imply an overprint as proposed by Lynch-Stieglitz et al.^41^. Any pronounced decrease in EP might also affect and increase both δ^13^C and [CO_3_^2−^]. In the South Atlantic, a reduction in EP coincided with a HS1 increase in [CO_3_^2−^], pointing to decreased biological productivity as a contributing factor to rising CO_2_^45^. A similar process could presumably have driven or contributed to both pulses observed during HS1 and the YD. The analysis of ^230^Th normalized fluxes of biogenic opal, carbonate, and excess barium on a suite of sediment records from the SW-Pacific indicate no pronounced change in EP since the LGM^25^. Thus, while a contribution of changes in EP cannot be excluded, we assume that it would only have a subordinate effect on the patterns observed. Given that AAIW could integrate signals from broad Southern Ocean regions due to homogenization by Antarctic Circumpolar Current, we want to encourage further work. Another factor to consider is the formation of AAIW and its subduction toward our core location. If the formation of low [CO_3_^2−^] AAIW is reduced, an increase in concentrations at our core site can be expected. The formation of AAIW is closely coupled to the applied wind stress^46^. During the glacial, stationary SW-Pacific ocean fronts^47^ in combination with a northward displacement of Southern Westerly Winds^24^, reduced the wind stress experienced in the formation area of SO AAIW. These processes in combination to changing salinity contrasts might have been coupled to the reduced the glacial subduction of SO AAIW (Ronge et al., 2015). Given these local effects, we expect only a subordinate role on the patterns recorded in PS75/104-1.

The HS1 pulse of increasing atmospheric CO_2_ was accompanied by the most dramatic drop of atmospheric δ^13^C as reconstructed Antarctic ice cores^3^ (Fig. 3f). Thus, the release of CO_2_ via the Southern Ocean, as implied by PS75/104-1 and Bay of Plenty records^39,40^, illustrates a likely mechanism that can account for the coevolution of atmospheric CO_2_ and δ^13^C-values (Fig. 3)^1,3^.

Throughout the ACR (14.5–12.9 ka)^48^, when atmospheric CO_2_ plateaued^1^ and its δ^13^C briefly returned to higher values^3^, intermediate-water [CO_3_^2−^] and δ^13^C return to lower, glacial-like values (Fig. 3). This suggests that expanding sea ice during the ACR disrupted the communication of upwelling deep waters with the atmosphere, before being incorporated into AAIW. These findings contrast ^14^C reconstructions from deep sea corals, in the South Tasman Sea bathed by AAIW^16^. The zonal asymmetry might be explained by regional differences in the movement of Southern Ocean fronts that are less constrained by sea floor topography in the Indo-Pacific sector^16^.

During the YD there is evidence in the ice cores for a second pulse of increasing CO_2_ and decreasing δ^13^C, this is again accompanied by an increasing intermediate-water [CO_3_^2−^] and δ^13^C in PS75/104-1 (Fig. 3). Thus, our AAIW record at PS75/104-1 points to a strong mechanistic link between Southern Ocean ventilation and atmospheric CO_2_ that was active in the SW Pacific.

However, to understand the importance of this Southern Ocean pathway of CO_2_ in the deglacial carbon system, we have to address several important questions: Which key regions contributed to the two-pulse, deglacial rise in atmospheric CO_2_^7,34,39,49–52^? What were the mechanisms and reservoirs that resulted in the release of CO_2_^10,11,53,54^, and which role did these have on the patterns observed in intermediate waters off New Zealand (PS75/104-1)?

In combination with shifting Southern Westerly Winds^24^, the deglacial retreat of Antarctic sea ice^29^ resulted in an intensification of upwelling of Circumpolar Deep Water throughout the Southern Ocean (Fig. 3e)^50^. Deep-water records from the Southern Ocean show significant perturbations during HS1 and the early deglacial. Radiocarbon values from the Atlantic, Indian, and Pacific Sectors of the Southern Ocean indicate an increase in deep-water ventilation that reflects the renewed contact of the deep and shallow overturning cells and thus exchange of the glacial carbon pool with surface waters and the atmosphere^5,7,8^. Simultaneously, a rise in CDW [CO_3_^2−^]^39,52^ and coral-derived δ^11^B^34^ reflect the loss of CO_2_ from this lower cell, coeval with increasing deep-water ventilation throughout HS1 (Fig. 3). Increasing pH in the lower overturning cell and decreasing pH in the upper cell indicate a release of CO_2_ from the deep Drake Passage during HS1^34^. However, our understanding of the lower cell carbon chemistry in other sectors of the Southern Ocean is still poorly constrained. In this respect, our AAIW data from the Pacific Sector suggest a close link between sea ice and westerly winds^24,29^, upwelling intensity^50^, deep-water ventilation and carbonate chemistry^8,39,52^, and atmospheric CO_2_ (Figs. 3 and 5). Intensified air-sea gas exchange triggered a transient increase in [CO_3_^2−^] in the formation area of AAIW. The signal from this process was subsequently exported from the formation area of SW Pacific AAIW^12^ toward the core location of PS75/104-1 (Figs. 1 and 5). During HS1, the abrupt reduction in Bay of Plenty intermediate-water Δδ^13^C (663–1165 m) suggests the loss of CO_2_ from SW Pacific AAIW^40^. Radiocarbon reconstructions from a southeasterly bathymetric transect off New Zealand likewise identified the ventilation from upwelling deep-waters in the formation area of Southern Ocean AAIW^8^. HS1 upwelling and ventilation of carbon-rich deep waters did not affect the (^14^C) ventilation of PS75/104-1^8^ (Fig. 4), while [CO_3_^2−^] in our record as well as nearby AAIW record RR0503-79^39^ show a significant excursion to higher values. Collectively this argues for a loss of CO_2_ in the formation area of AAIW.

In combination with other Southwest Pacific records that display a similar HS 1 pattern^8,15,17,39,52^ our AAIW data highlight the importance the Southern Ocean’s Pacific pathway had on the HS1 atmospheric CO_2_ increase. The majority of AAIW reaching our core location is formed directly to the south as so called SO AAIW^12^. Nevertheless, given the similar pattern evident in the Bay of Plenty records^39^ it could also be due to an upstream contribution from the SE Pacific (the primary formation region of AAIW) or the Indo Pacific via the Antarctic Circumpolar Current^7,16^.

Following HS1, the southern hemispheric ACR was marked by a reduction in Southern Ocean upwelling rates, however less pronounced than during the LGM (Fig. 3e)^50^. In PS75/104-1 [CO_3_^2−^] and δ^13^C values rapidly decreased during the ACR (Fig. 2). The decrease in opal flux (suggested to be an indicator of upwelling of carbon-rich deep-waters)^50^ and an enhanced winter and spring sea ice cover^55^, reduced the carbon loss in the formation area of AAIW. Throughout the Austral summer and autumn, ACR sea-ice and biological feedbacks increased the sequestration of CO_2_ in the high southern latitudes^55^. In combination, these factors provide a likely scenario as the mechanism for the ACR trends seen in our record and highlight the fact that AAIW off New Zealand (PS75/104-1) can trace upstream changes in the Antarctic Zone.

Following the ACR, during the YD AAIW [CO_3_^2−^] (this study) and opal flux suggest a reinvigorated upwelling in the Antarctic zone of the Southern Ocean (Fig. 3e)^50^. This is supported by other records from the Southern Ocean. In the South Atlantic, two records from 4276 m^56^ and 4981 m^57^ point toward a progressive deepening in the erosion of the deep ocean carbon pool. Similar to the South Atlantic^57^, it is likely that the ventilating water masses came from below ~ 4300 m. Radiocarbon-based reconstructions of deep-water ventilation show that down to this depth, deep-water ventilation reached modern-like values at the end of HS1^8^. There is also evidence for a Southern Ocean contribution to the second pulse in atmospheric CO_2_ from rapidly decreasing pH values of the lower cell in the Drake Passage (Fig. 3d)^34^, and the Southern Indian Ocean off the Kerguelen Archipelago where steepening isohalines and isopycnals decreased stratification and allowed for a resumption of deep-water ventilation^7^. Other records of deep- and intermediate water [CO_3_^2−^]^39,52^, and radiocarbon^8^ from the SW Pacific, lack sufficient resolution across the YD. Our study provides more data points and significantly improved chronological constraints during this time interval^13^.

Thus, while the main part of the atmospheric CO_2_-increase during the YD is thought to be due to the thawing permafrost soils on the northern hemisphere^10,11^, our data can now point to southern hemispheric contribution of CO_2_ at this time via outgassing from the Southern Ocean (Fig. 5b). During the Holocene AAIW records RR0503-79^39^ and PS75/104-1 begin to diverge (Fig. 3). The youngest values of both records agree with modern [CO_3_^2−^] data^39,58^, likely the result of two different sources and pathways of AAIW at the core sites; Tasman AAIW at the Bay of Plenty site, and SO AAIW at our Chatham Rise site, as defined by Bostock et al.^12^.

During the last deglacial, the history of AAIW [CO_3_^2−^] and δ^13^C in our record closely trace Southern Ocean upwelling rates from opal flux^50^, as well as atmospheric δ^13^C^3^ and CO_2_^1^ (Fig. 3). Between ~ 18 and 11 ka, changes in the extent of Antarctic sea ice cover^59^ and the meridional shift of the southern westerly winds^54^ modulated the upwelling rates of CDW^50^. In combination with changes in the efficiency of the biological carbon pump^53^, the increased communication of CO_2_-rich deep-waters via Southern Ocean upwelling was the main driver of early deglacial atmospheric CO_2_. Both transient peaks, observed in PS75/104-1 during HS1 and the YD, are indicative of a loss in CO_2_^14,27^ in the formation area of AAIW. The very close relationship between AAIW [CO_3_^2−^] and δ^13^C with atmospheric patterns (Figs. 3 and 5) highlights the Southern Oceans role on the deglacial climate. Deglacial deep-water records of Bay of Plenty [CO_3_^2−^]^39^ and δ^13^C^17^ (Figs. 3c and 6), and Bounty Trough ΔΔ^14^C^8^ (Fig. 4) indicate a progressive change in water mass properties, indicative of circulation induced shifts in water mass mixing and/or loss of respired CO_2_ through ventilation^39^.

During time periods with cold northern hemispheric stadial conditions (HS1 or YD), the bipolar seesaw hypothesis^60^ argues for a reduction in the efficiency of the AMOC. A diminished AMOC results in the reduced export of heat from the southern hemisphere to the northern hemisphere, ultimately triggering a decrease in Antarctic sea ice that contributed to Southern Ocean release of CO_2_. The warmer northern hemispheric Bølling-Allerød period again resulted in a strengthening of North Atlantic Deep Water formation and the AMOC^61^. This period roughly correlates to the ACR that saw an increase in Antarctic sea ice, a northward displacement of southern westerly winds and reduced upwelling of CDW^50^. As our data show, the ACR was also marked by a return to glacial-like [CO_3_^2−^] and δ^13^C (Fig. 3) and thus reduced air-sea gas exchange in the Southern Ocean, the area of AAIW formation. The well constrained temporal evolution of AAIW [CO_3_^2−^] and δ^13^C throughout the entire deglacial period (Fig. 3) provides important new insight into the key role, the Southern Ocean played in the two-step rise of atmospheric CO_2_.

## Conclusions

Our investigation of foraminifer-based [CO_3_^2−^] and δ^13^C records on an intermediate water core off New Zealand highlight the role SW-Pacific AAIW played during the deglacial rise in atmospheric CO_2_. In conclusion, we propose that:

1. Reconstructed [CO_3_^2−^] and δ^13^C trends point to a potential release of respired CO_2_ through ventilation in the upwelling region of circumpolar deep water.
2. Our findings agree with previous studies from the region that indicated that the mid-depth Pacific acted as a reservoir for CO_2_ during the last glacial^8,39,40,52,62^.
3. The observed transient rises in [CO_3_^2−^] and δ^13^C during HS1 are consistent with the release of CO_2_ during this interval. While this interpretation is not unambiguous, it adds to a growing set of studies that indicate a similar process^8,39,40,50,52,63^.
4. In addition to northern hemisphere sources^10,11^, the YD rise in atmospheric CO_2_, might have experienced a contribution of released CO_2_ from the South Pacific as well.
5. Throughout the Holocene, AAIW δ^13^C probably experienced an overprint from atmospheric values^41^.
6. *C. dispars* can be used for reconstructions, using our new calibration B/Ca = 2.27(Δ[CO_3_^2−^]) + 152.37

## Materials and methods

### Sediments and sample treatment

We analyzed sediment core PS75/104-1 that was retrieved during expedition ANTXXVI/2 at S44° 46′ 9.012′′ E174° 31′ 31.8′′ in a water depth of 835 m (AAIW), using a BGR type piston corer. The core was split into an archive and a working half and subsequently sampled. All samples were frozen and freeze dried for 2–3 days. The dried samples were wet-sieved, using a 63 µm mesh sieve and subsequently dried at 50 °C for 2–3 days. The > 63 µm fraction was subdivided into the size fractions < 125 µm, 125–250 µm, 250–315 µm, 315–400 µm, and > 400 µm. Planktic and benthic foraminifers used were picked from the 250–315 and 315–400 µm fractions.

### Age control

For the core interval between 0 and 0.18 m, the age model for PS75/104-1 is based on the results of Ronge et al.^8^. Below 0.18 m, we used the new age model provided by Küssner et al.^13^, based on the highly accurate plateau tuning technique and 56 planktic ^14^C ages. Thus, we have a very reliable age control on the deglacial interval discussed in this study (7.4–17.8 ka). Throughout this time interval, sedimentation rates range between 16 and 32 cm/ky^13^. Some depths of PS75/104-1 might be affected by pronounced *Zoophycos* burrows that were mapped by densely spaced ^14^C-samples and X-radiographies^13^. However, only four of our samples fall into these intervals (Fig. 2). Excluding these would not affect any of our interpretations. Hence, we are highly confident in the integrity of our records.

### Stable carbon isotopes

To determine past water mass δ^13^C values we analyzed monospecific samples (2–4 specimens) of *Cibicidoides wuellerstorfi*. The measurements were conducted at the Alfred Wegener Institute Helmholtz Center for Polar and Marine Research in Bremerhaven, using Finnigan MAT 253 and 251 spectrometers, coupled to carbonate preparation devices Kiel II and Kiel IV, respectively. Based on an internal laboratory standard (Solnhofen limestone), the long-term precision over one year was better than 0.06 ‰. Isotope ratios are reported as ‰ deviations (δ) from the Vienna PeeDee Belemnite (VPDB) standard.

### B/Ca measurements and [CO_3_^2−^] calculation

**Figure 7:**
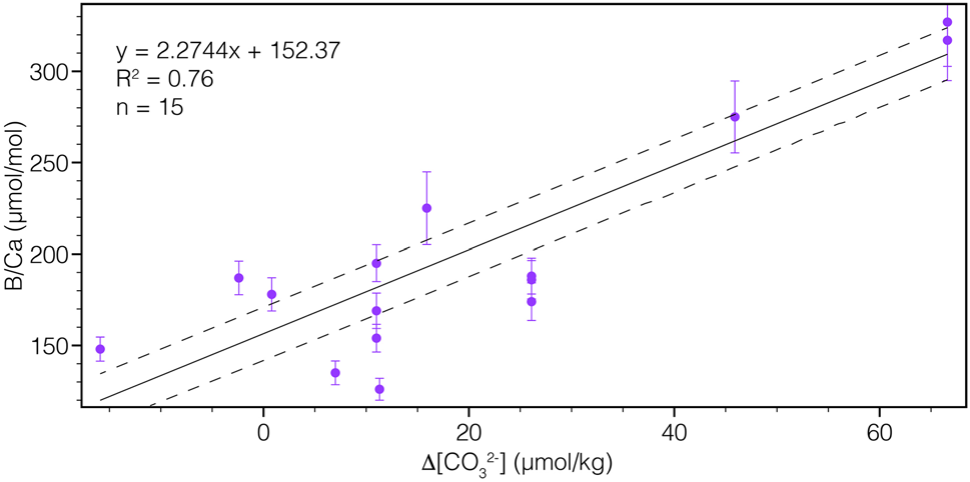
Calibration of core-top B/Ca (1σ-error bars) against pre-industrial water mass Δ[CO_3_^2−^]^6^. Dashed lines show the ± 15 µmol/mol uncertainty envelope, calculated according to Yu and Elderfield^70^.

B/Ca measurements were conducted on the 315–400 µm fraction of specimens of the epibenthic foraminifer species *Cibicidoides wuellerstorfi* and *Cibicidoides dispars*^64^, which showed no sign of alteration or secondary fillings. B/Ca analyses were conducted at the GEOMAR Helmholtz Center for Ocean Research in Kiel, using a Coherent GeoLasPro 193 nm Excimer laser ablation system, coupled to a Nu Instruments AttoM magnetic sector mass spectrometer. LA-ICP-MS is a well-established method for the analysis of foraminiferal calcite^65–67^. The analytical method we used for this study has been proven to be accurate and precise^68^ (instrument details given in the supplementary information). For each sample 3–6 specimens were analyzed on four 90 µm spots in the three oldest chambers on the umbilical side. Before and after each set of five specimens, the NIST615 standard^69^ was measured and used for calibration. Before beginning the analyses, each shell as well as the NIST615 standard were pre-ablated to prevent any surface contamination effects. Samples with ratios of Mn/Ca > 0.2 mmol/mol and Al/Ca > 0.4 mmol/mol were discarded from the dataset. For our calculations, we applied the calibration of Yu et al.^56^: B/Ca = 1.14(Δ[CO_3_^2−^]) + 176.6 for *C. wuellerstorfi*. For *C. dispars*, provide a new calibration B/Ca = 2.27(Δ[CO_3_^2−^]) + 152.37 (Supplementary text and Figs. 7 and S2). Modern [CO_3_^2−^]_sat_ of 49.48 µmol/kg was derived from GLODAP v2^58^. [CO_3_^2−^]_sat_ can be affected by changes in salinity (S), bottom water temperature (BWT), and pressure (P). Based on Yu and Elderfield^70^, we assume an insignificant impact of glacial-interglacial changes in S, BWT, or P on our downcore [CO_3_^2−^]. While probably more pronounced in intermediate-waters, than deep-waters, changes in S, BWT, and P are within the uncertainty of the proxy’s calibration^39,52^.

## Supplementary Information

Supplementary Information.
