# Supplementary material for: Southern Ocean contribution to both steps in deglacial atmospheric CO2 rise
Source: Sci Rep. 2021 Nov 11;11:22117. doi: 10.1038/s41598-021-01657-w (PMC8585946; doi:10.1038/s41598-021-01657-w)
Supplement: Supplementary file 1 — Supplementary Information. [file 41598_2021_1657_MOESM1_ESM.docx]

Supporting Information for

**Southern Ocean contribution to both steps in deglacial atmospheric CO_2_ rise**

Thomas A. Ronge, Matthias Frische, Jan Fietzke, Alyssa L. Stephens, Helen Bostock, Ralf Tiedemann

The supporting information contains instrument details, as well as details and calculations of the *Cibicidoides dispars* calibration, used in the main manuscript. For the calibration of *C. wuellerstorfi*, please refer to Yu et al.^1^.

**LA and ICP-MS operating parameters**

**Coherent GeoLas Pro excimer laser ablation system equipped with the manufacturer's standard ablation cell**

Laser wavelength: 193 nm (ArF excimer laser)

Laser pulse length: 15 ns

Fluence: 2J/cm^2^ (sample), 5 J/cm2 (NIST615)

Laser repetition rate: 2 Hz (sample), 10 Hz (NIST615)

Ablation spot size and geometry: 90 µm, round

Carrier gas flow: 0.70 – 1.03 l/min Helium

Transport gas flow: 0.70 – 1.06 l/min Argon

Ablation under pure Helium carrier gas, Argon transport gas was added after the ablation cell.

**Nu Instruments AttoM HR-ICP-MS**

RF power: 1000 – 1100 W

Analyser pressure: 2.0 – 4.1 x 10^-7^ mbar

Sample and skimmer cone material: Nickel

Plasma coolant gas flow: 13.5 – 14.0 l/min Argon

Auxiliary gas flow: 0.70 – 1.20 l/min Argon

Data acquisition: 5 sweeps per cycle (1.1 s)

Detector mode: linked mode (fast magnet scan with electrostatic deflection, ion counter, automatic attenuation)

**Data evaluation**

Data evaluation has been performed similar to the linear regression slope method described by Fietzke et al.^2^ using the INDEX(LINEST(.. .)) function of MS EXCEL.

For the calibration, we used core-top samples recovered from the South Pacific during expeditions PS75^3^ and SO213^4^ (Fig. S1A).

B/Ca measurements were conducted on the 315-400 µm fraction of specimens of the epibenthic foraminifer species *Cibicidoides dispars*^5^, which showed no sign of alteration or secondary fillings. B/Ca analyses were conducted at the GEOMAR Helmholtz Center for Ocean Research in Kiel, using a Coherent GeoLasPro 193nm Excimer laser ablation system, coupled to a Nu Instruments AttoM magnetic sector mass spectrometer^6^. For each sample 3-6 specimens were analyzed on four 90µm spots in the three oldest chambers on the umbilical side. Before and after each set of five specimens, the NIST615 standard^7^ was measured and used for calibration. Before beginning the analyses, each shell as well as the NIST615 standard were pre-ablated to prevent any surface contamination effects. Samples with ratios of Mn/Ca >0.2 mmol/mol and Al/Ca >0.4 mmol/mol were discarded from the dataset.

Analyzed B/Ca ratios of *C. dispars* range between 126 and 327 µmol/mol (Fig. S2B). Following the approach of Yu et al.^1^ for *C. wuellerstorfi*, we find a linear regression function of *C. dispars* B/Ca and water mass D[CO_3_^2-^] (Olsen et al., 2016)^8^, expressed as:

B/Ca = 2.27(Δ[CO_3_^2-^]) + 152.37 R^2^ = 0.76 n=15

**Supplementary References**

1 Yu, J. *et al.* Deep South Atlantic carbonate chemistry and increased interocean deep water exchange during last deglaciation. *Quaternary Science Reviews* **90**, 80-89 (2014).

2 Fietzke, J. *et al.* An alternative data acquisition and evaluation strategy for improved isotope ratio precision using LA-MC-ICP-MS applied to stable and radiogenic strontium *Journal of Analytical Atomic Spectrometry* **23** (2008).

3 Gersonde, R. The Expedition of the Research Vessel "Polarstern" to the polar South Pacific in 2009/2010 (ANT-XXVI/2 - BIPOMAC). (Alfred Wegener Institute, Bremerhaven, 2011).

4 Tiedemann, R. FS Sonne Fahrtbericht / Cruise Report SO213. (Alfred Wegener Institute, Bremerhaven, 2012).

5 Hayward, B. W., Grenfell, H. R., Sabaa, A. T., Neil, H. L. & Buzas, M. A. *Recent New Zealand deep-water benthic foraminifera: Taxonomy, ecologic distribution, biogeography, and use in paleoenvironmental assessment*. 278-343 (GNS Science, 2010).

5 Fietzke, J. & Frische, M. Experimental evaluation of elemental behavior during LA-ICP-MS: influences of plasma conditions and limits of plasma robustness. Journal of Analytical Atomic Spectrometry 31, 234-244 (2016).

7 Jochum, K. P. *et al.* Determination of Reference Values for NIST SRM 610--617 Glasses Following ISO Guidelines. *Geostandards and Geoanalytical Research* **35**, 397-429 (2011).

8 Olsen, A. *et al.* The Global Ocean Data Analysis Project version 2 (GLODAPv2) – an internally consistent data product for the world ocean *Earth System Science Data* **8**, 297-323 (2016).

9 Lynch-Stieglitz, J., Stocker, T. F., Broecker, W. S. & Fairbanks, R. G. The influence of air-sea exchange on the isotopic composition of oceanic carbon: Observations and modeling. *Global Biogeochemical Cycles* **9**, 653-665 (1995).

10 Stott, L. *et al.* CO_2_ Release From Pockmarks on the Chatham Rise-Bounty Trough at the Glacial Termination. *Paleoceanography and Paleoclimatology* **34**, PA003674 (2019).

11 Yu, J. & Elderfield, H. Benthic foraminiferal B/Ca ratios reflect deep water carbonate saturation state. *Earth and Planetary Science Letters* **258**, 73 (2007).

**Supplementary Figures**


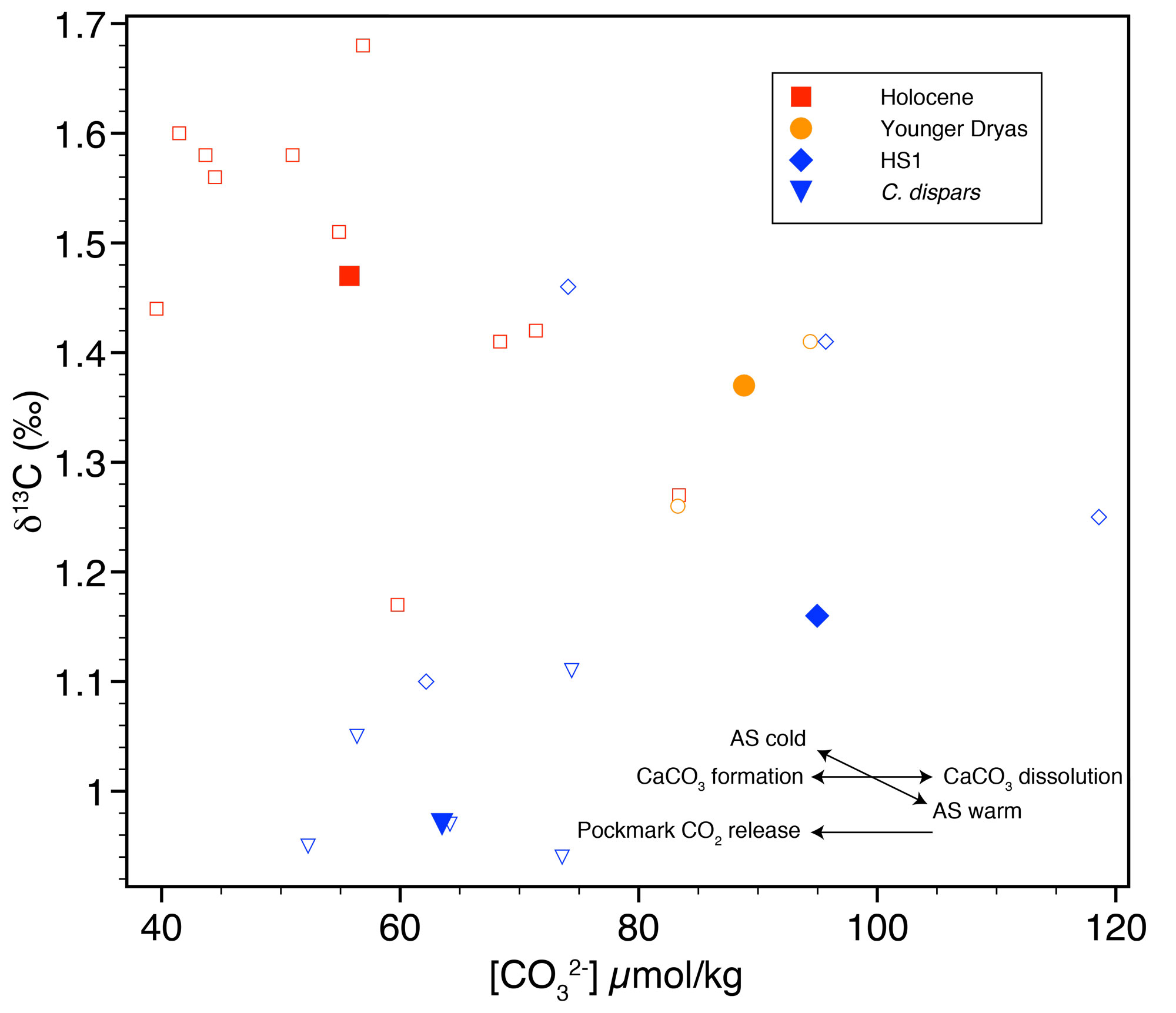


**Figure S1:** Cross-plot of PS75/104-1 [CO_3_^2-^] and δ^13^C. Filled symbols – mean values; empty symbols individual data points. AS – Air-sea gas exchange, slope after (Lynch-Stieglitz et al.^9^; pockmark line estimated after Stott et al.^10^.


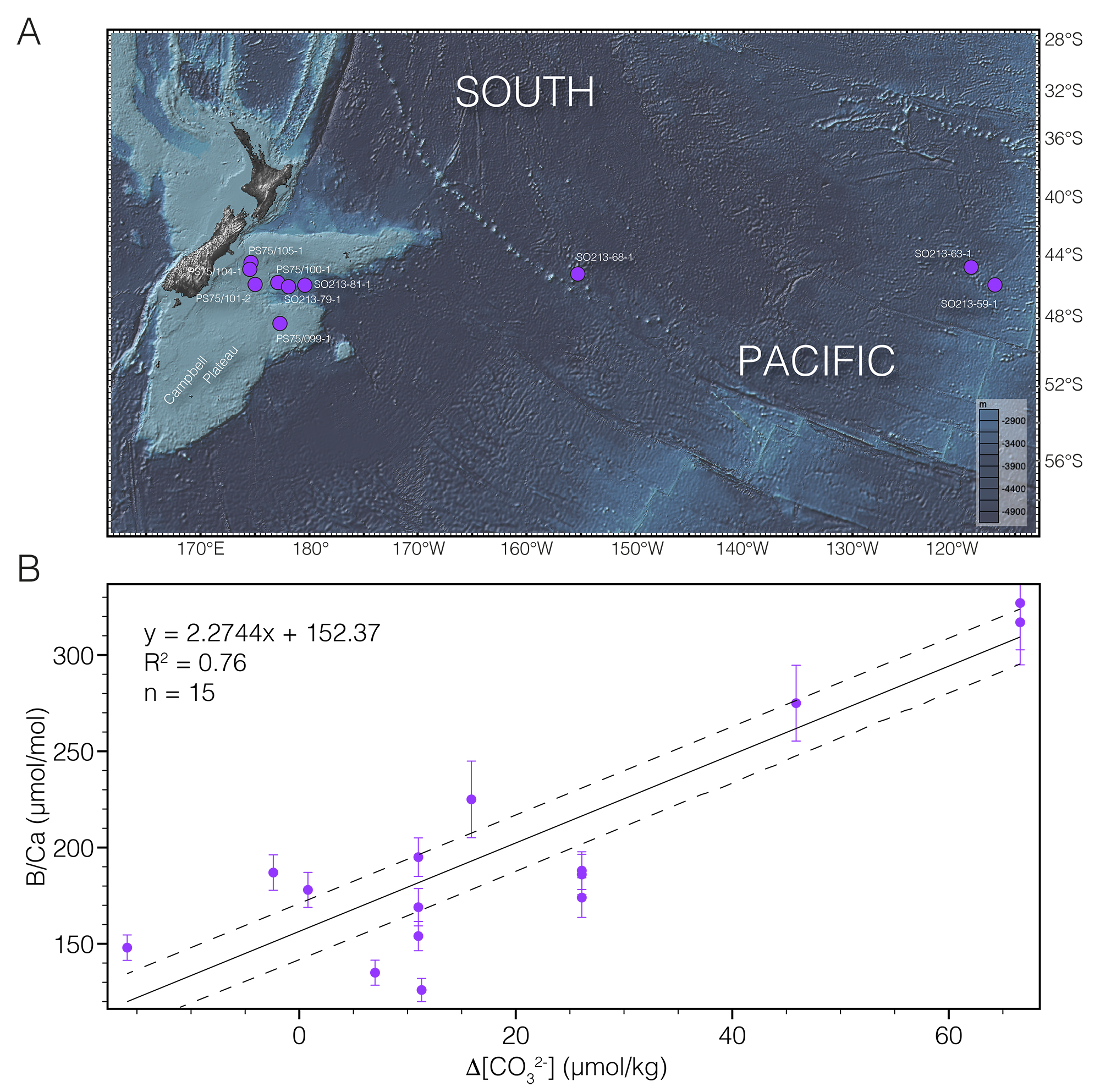


**Figure S2: (A)** Locations of core-top samples used for the calibration. **(B)** Calibration of core-top B/Ca (1σ-error bars) against pre-industrial water mass Δ[CO_3_^2-^]^Ref.8^. Dashed lines show the ±15 µmol/mol uncertainty envelope calculated according to Yu and Elderfield^11^. Map generated with GeoMapApp 3.6.12 (www.geomapapp.org). Data archived at https://doi.pangaea.de/10.1594/PANGAEA.931987.
